# The Driving Force for 2014 Dengue Outbreak in Guangdong, China

**DOI:** 10.1371/journal.pone.0166211

**Published:** 2016-11-18

**Authors:** Ming-Tao Li, Gui-Quan Sun, Laith Yakob, Huai-Ping Zhu, Zhen Jin, Wen-Yi Zhang

**Affiliations:** 1 Complex Systems Research Center, Shanxi University, Taiyuan, Shan’xi, People’s Republic of China; 2 School of Computer and Information Technology, Shanxi University, Taiyuan, Shan’xi, People’s Republic of China; 3 Department of Disease Control, London School of Hygiene and Tropical Medicine, London, United Kingdom; 4 LAMPS and Department of Mathematics and Statistics, York University, Toronto, Canada; 5 Institute of Disease Control and Prevention, Academy of Military Medical Science, Beijing, People’s Republic of China; Lanzhou University of Technology, CHINA

## Abstract

Dengue fever has rapidly spread in recent decades to become the most globally expansive viral vector-borne disease. In mainland China, a number of dengue outbreaks have been reported since 1978, but the worst epidemic in decades, involving 45230 cases and 76 imported cases, resulting in six deaths in Guangdong province, emerged in 2014. Reasons for this ongoing surge in dengue, both imported and autochthonous, are currently unclear and demand urgent investigation. Here, a seasonally-driven dynamic epidemiological model was used to simulate dengue transmission data recorded from the unprecedented outbreak. Sensitivity analysis demonstrate that delayed mosquito control, the continuous importations between the end of April to the early of July, the transmission of asymptomatic dengue infections, and the abnormally high precipitation from May to August might be the causal factors for the unprecedented outbreak. Our results suggested that the earlier and more frequent control measures in targeting immature and adult mosquitoes were effective in preventing larger outbreaks, and enhanced frontier health and quarantine from the end of April to the early of July for international communications and travelers.

## Introduction

Dengue is a mosquito-borne viral infection causing a severe flu-like illness, and sometimes causing a potentially lethal complication called severe dengue [1]. In recent decades, the significance of dengue as a threat to health and a burden on health services and economies has increased substantially. Almost half the world’s population lives in at risk regions for dengue virus transmission, and the World Health Organization (WHO) estimates that 50–100 million dengue infections occur annually in over 100 endemic countries in Africa, America, Southeast Asia and the Western Pacific. More than 70% of people at risk reside in the Asia Pacific region, making this region the global epicenter of dengue activity [2]. Dengue can be transmitted by the bite of a female mosquito infected with one of the four dengue virus serotypes. The primary vector are the *Aedes aegypti* and *Aedes albopictus* mosquito which mostly thrive in urban and semi-urban areas with tropical or sub-tropical climates [3]. Currently, the method to control or prevent the transmission of dengue virus is through vector management [4] which is often logistically difficult, and which has demonstrated considerable variability in effectiveness. Hence, new insights and tools to improve public health system preparedness are of increasingly high priority [5, 6].

In 1978, an outbreak in Foshan Guangdong province signalled the reemergence of dengue in mainland China after being absent for 30 years [7]. Dengue became a nationally notifiable disease on 1 September 1989; and all cases of dengue fever were diagnosed according to the unified diagnostic criteria issued by the National Health and Family Planning Commission, which includes definitions of clinically diagnosed and laboratory-confirmed cases [8]. Although dengue epidemics have frequently occurred since the 1990s, dengue fever is still characterized as an imported epidemic disease and has not yet been confirmed to be endemic in mainland China [9]. However, with the rapid growth of the Chinese economy, international travel, particularly between Southeast Asia and China, importation of people traveling is more frequent than ever. This human movement creates major challenges in preventing and controlling the spread of non-endemic infectious diseases.

Guangdong province lies in southeastern China, characterized by a humid subtropical climate, where the *Aedes albopictus* mosquitos are widely distributed and regarded as the sole vector for dengue transmission [10–13]. Dengue cases reported for mainland China and specifically Guangdong province from 1990 to 2013 are shown in Table 1. Guangdong province has the highest incidence of dengue cases in mainland China, with frequent, sporadic epidemics sparked from imported infections [14]. In 2014, an unexpectedly large dengue epidemic was reported in Guangdong, involving 45230 cases and 76 imported cases in Guangdong province, and exceeding the cumulative number of cases from 1990 to 2013. Understanding the factors influencing modern dengue outbreaks in Guangdong has become a major national public health priority.

**Table 1:** Comparison dengue cases between mainland China and Guangdong from 1990 to 2013. The mainland China data are from Refs. [8] and the Guangdong data are from Refs. [9, 13].

Year	1990	1991	1992	1993	1994	1995	1996	1997	1998	1999	2000	2001
Mainland China	376	902	2	367	4	6836	2	634	490	1868	405	375
Guangdong province	374	371	2	359	4	6812	2	632	480	290	401	365
Year	2002	2003	2004	2005	2006	2007	2008	2009	2010	2011	2012	2013
Mainland China	1606	93	247	59	1063	551	254	322	260	160	610	4779
Guangdong province	1576	82	49	23	1010	397	87	19	139	49	474	2894

Mathematical models can provide useful strategic insights into control measures for infectious diseases [15–17]. Several dynamic models of dengue have been published in recent years and have proven useful in informing vector control strategies which target either immature or mature mosquito stages [18–25]. Based on temperature-controlled mosquito experiments of Yang et al. [25], a series of theoretical analysis were published utilizing these novel data to inform more flexible approaches to understanding temperature effects on various life history traits [26–30]. In recent two years, some researches on 2014 Guangzhou outbreak data (only including the symptomatic data) were published [31–34]. Sang et al. [31, 32] claimed that the number of imported cases, minimum temperature with a one-month lag and cumulative precipitation with a three month lag predicted the outbreak in 2013 and 2014 by using a multivariate Poisson regression analysis of the Guangzhou outbreak data. Cheng et al. [33] used a mathematical model to obtain that climate and the timing of imported cases as the causal factors of dengue outbreak in Guangzhou (The authors assumed that only one case was imported to Guangzhou in the model, this assumption was actually wrong). Zhu et al. [34] found that urbanization, vector activities, and human behavior play significant roles in shaping the dengue outbreak and the patterns of its spread by using a spatio-temporal patterns model. In recent study, Chastel [35] concluded that asymptomatic dengue infections could cause new foci of disease or eventually an epidemic in non-endemic regions. In fact, it is difficult to address the real number of infected dengue fever cases, since most of them are asymptomatic [3, 35, 36], and low dengue reporting rates have previously been found in South Asia and Southern China [33, 34, 37, 38]. As a result, the transmission of asymptomatic dengue virus infections was ignored in most studies.

In this paper, to investigate the causal factor for 2014 dengue outbreak of Guangdong, we developed a seasonally-driven dynamic epidemiological model of dengue transmission between human and mosquito hosts that takes into account the transmission both imported dengue cases and asymptomatically infected cases. Then the parameters in the model were estimated, and numerical simulations support the data reasonably well. Finally, sensitivity analyses are conducted to investigate the causal factor for the unprecedented outbreak of dengue in 2014 in Guangdong.

## Materials and Methods

### The 2014 dengue outbreak of Guangdong, China

**Fig 1:**
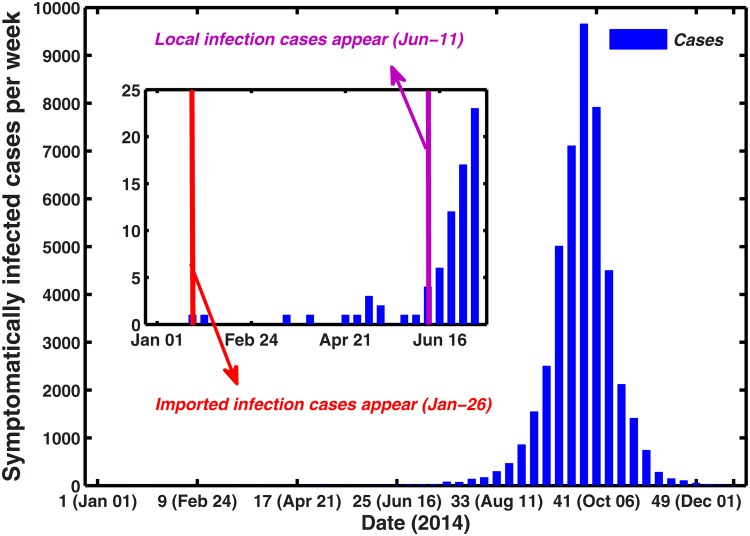
Number of dengue cases reported in Guangdong province, China, 2014.

**Fig 2:**
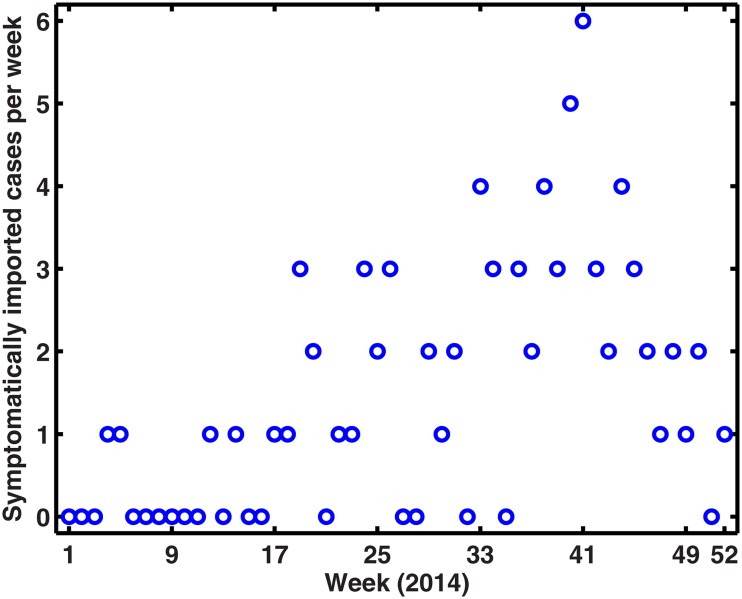
The weekly imported dengue cases in Guangdong, 2014.

**Fig 3:**
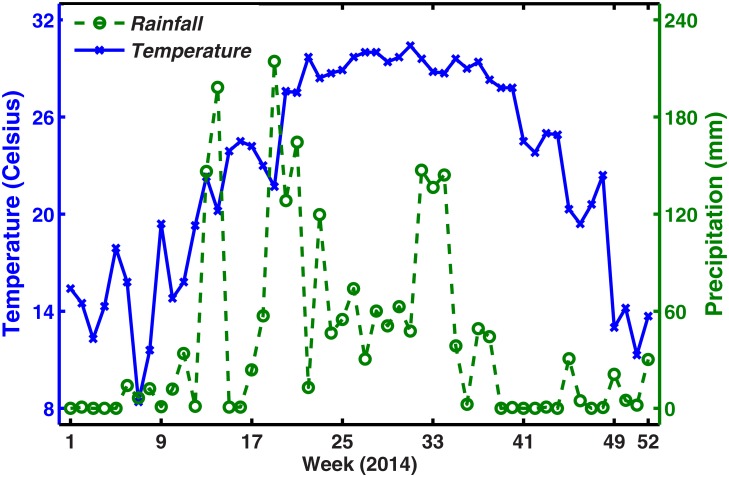
Climate for Guangdong province from January-2014 to December-2014. Weekly average temperatures (solid, blue), and weekly precipitation (dotted, green).

During the 25-year period from 1990 to 2014, 69,321 cases of dengue including 11 deaths were reported to the national dengue surveillance system in mainland China [8]. A major outbreak in 2014 constituted most of this total including 47056 dengue cases, 45230 of which were in Guangdong province. Dengue is a nationally notifiable disease in China—physicians must report all diagnosed cases to the China Center for Disease Control and Prevention through the China Information System for Disease Control and Prevention (CISDCP). Fig 1 shows the symptomatically reported dengue case data for Guangdong province per week in 2014. The 2014 dengue outbreak in Guangdong presents a sharp initial rise in the number of reported cases and an equally fast decline towards the end of the epidemic. The first symptomatically imported dengue infection (The infection had recent overseas travel history recorded) was reported on 26 January (the fourth week), and the first symptomatically autochthonous case was not recorded until 11 June (the 24th week) 2014. The weekly imported dengue cases for Guangdong province in 2014 is shown in Fig 2, which mainly contains international communications and travelers from Southeast Asia. Fig 3 shows the weekly temperature and precipitation data for Guangzhou. We use climate data for Guangzhou, given the city’s predominant role in this outbreak with approximately 83% of the total cases [33].

### The Mathematical Model

**Fig 4:**
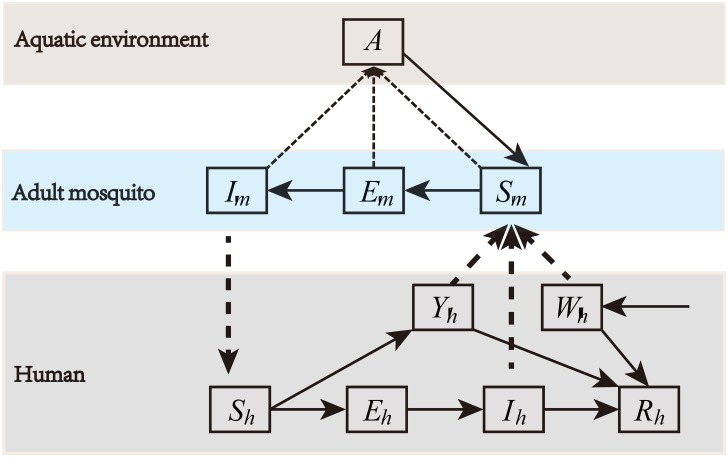
The transmission of dengue virus between mosquitoes and humans. Black dash-dotted lines show direction of transmission between humans and mosquitoes. For mosquito, *A* denotes immature stage, and *M* describes mature mosquito stage (including susceptible *S*_*m*_, incubating *E*_*m*_, and infectious *I*_*m*_), and *M* = *S*_*m*_ + *E*_*m*_ + *I*_*m*_. For human, all hosts are divided into six stages for dengue transmission: susceptible *S*_*h*_, incubating *E*_*h*_, asymptomatic infectious *Y*_*h*_, infectious *I*_*h*_, imported infectious *W*_*h*_ and recovered *R*_*h*_, where *N*_*h*_ = *S*_*h*_ + *E*_*h*_ + *Y*_*h*_ + *I*_*h*_ + *W*_*h*_ + *R*_*h*_ is the total human population.

Fig 4 shows a flow diagram to describe the transmission dynamics of dengue virus between humans and mosquitoes (which also calls vector population). For the dynamics of the vector population, the mosquito hosts can be divided into immature stage *A* (eggs, larvae and pupae) and mature mosquito stage *M* by using the study of Yang et al. [25]. Their population dynamics are described by the following ordinary differential equations:

$$\left\{\begin{array}{c} \frac{dA}{dt}=kf\theta \left(T\right)M-\frac{A^{2}}{\kappa (t)}-\left(\mu_{a}\left(T\right)+\varepsilon \left(T\right)\right)A,\\ \frac{dM}{dt}=\varepsilon \left(T\right)A-\mu_{m}\left(T\right)M, \end{array}\right.$$ (1)

where *θ*(*T*) is the intrinsic oviposition rate of an adult mosquito, *κ*(*t*) denotes the inhibition rate of precipitation on larva, *μ*_*a*_(*T*) and *μ*_*m*_(*T*) are the respective mortality rate of immature forms and adult mosquito, *ε*(*T*) is the transition rate from immature stages into adult mosquitoes. Eggs do not all hatch into larvae, nor do they all produce female mosquitoes. For these reasons, *k* is the fraction of eggs hatching to larvae, and *f* is the fraction of female mosquitoes hatched from all eggs.

We further extend the adult female class by subdivision into the epidemiologically relevant stages for dengue transmission: susceptible *S*_*m*_, incubating *E*_*m*_, and infectious *I*_*m*_. The incubation period is *σ*_*m*_(*T*) days; *b*(*T*) is the biting rate and *β*_*mh*_ is the infected human to susceptible mosquito (human-to-vector) transmission probabilities per bite. The human population is assumed to be fully susceptible to the virus. For humans, upon challenge with infectious mosquito bites, susceptible humans become exposed at a rate *p*, and become asymptomatic infectious with a rate 1 − *p*. *β*_*hm*_ is the infected mosquito to susceptible human (vector-to-human) transmission probability per bite. The human host incubation period is *σ*_*h*_ days, the duration of symptomatic infectious, asymptomatic infectious and imported infectious are *γ*_*h*_, *γ*_*y*_ and *γ*_*w*_ days, respectively. Recovery is assumed to yield life-long immunity. For the imported infection *W*_*h*_ compartment, *B*(*t*) and $\frac{1-p}{p}B\left(t\right)$ are the recruitment rate of symptomatically and asymptomatically infected individuals at time *t*. The human mortality was ignored for this model.

In Guangdong, on every Friday afternoon from September 24th to late November in 2014, insecticidal fogging and mosquito repellents were used to kill mature mosquitos with a fraction *α*_*m*_; Water containers were also emptied to remove immature mosquitos and destroy their breeding sites with a fraction *α*_*A*_ [33].

Hence, the following system of equations can be used to describe dengue transmission:

$$\left\{\begin{array}{c} \frac{dA}{dt}=kf\theta \left(T\right)M-\frac{A^{2}}{\kappa (t)}-\left(\mu_{a}\left(T\right)+\varepsilon \left(T\right)\right)A-\alpha_{A}A,\\ \frac{dS_{m}}{dt}=\varepsilon \left(T\right)A-b\left(T\right)\beta_{hm}\frac{S_{m}\left(Y_{h}+I_{h}+W_{h}\right)}{N_{h}}-\mu_{m}\left(T\right)S_{m}-\alpha_{m}S_{m},\\ \frac{dE_{m}}{dt}=b\left(T\right)\beta_{hm}\frac{S_{m}\left(Y_{h}+I_{h}+W_{h}\right)}{N_{h}}-\left(\frac{1}{\sigma_{m}\left(T\right)}+\mu_{m}\left(T\right)\right)E_{m}-\alpha_{m}E_{m},\\ \frac{dI_{m}}{dt}=\frac{1}{\sigma_{m}\left(T\right)}E_{m}-\mu_{m}\left(T\right)I_{m}-\alpha_{m}I_{m},\\ \frac{dS_{h}}{dt}=-b\left(T\right)\beta_{mh}\frac{S_{h}I_{m}}{N_{h}},\\ \frac{dE_{h}}{dt}=pb\left(T\right)\beta_{mh}\frac{S_{h}I_{m}}{N_{h}}-\frac{1}{\sigma_{h}}E_{h},\\ \frac{dY_{h}}{dt}=\left(1-p\right)b\left(T\right)\beta_{mh}\frac{S_{h}I_{m}}{N_{h}}-\frac{1}{\gamma_{y}}Y_{h},\\ \frac{dI_{h}}{dt}=\frac{1}{\sigma_{h}}E_{h}-\frac{1}{\gamma_{h}}I_{h},\\ \frac{dW_{h}}{dt}=B\left(t\right)+\frac{1-p}{p}B\left(t\right)-\frac{1}{\gamma_{w}}W_{h},\\ \frac{dR_{h}}{dt}=\frac{1}{\gamma_{h}}I_{h}+\frac{1}{\gamma_{y}}Y_{h}+\frac{1}{\gamma_{w}}W_{h},\\ \frac{dC_{h}}{dt}=\frac{1}{\sigma_{h}}E_{h}+B\left(t\right), \end{array}\right.$$ (2)

where *C*_*h*_ is included to track the cumulative number of dengue infections. The newly infected cases between *t* − 1 and *t* are expressed as follows,

$$X\left(t\right)=C_{h}\left(t\right)-C_{h}\left(t-1\right).$$

To emphasize local characteristics in Guangdong, our model included local control strategies, the transmission of imported infectious and asymptomatic infectious, which are absent from other models.

### Temperature-dependent parameters

Based on experiments by Yang et al. [25] on *Aedes aegypti* mosquitoes over the temperature range of 10.54°*C* ≤ *T* ≤ 33.41°*C*, we also use the expressions of the intrinsic oviposition rate *θ*(*T*), the mortality rate of aquatic forms *μ*_*a*_(*T*) and adult mosquitoes *μ*_*m*_(*T*), as well as the transition rate from pupae into adults *ε*(*T*) for *Aedes albopictus* in Guangdong, China. Here, *T* is temperature in Celsius.

**The intrinsic oviposition rate** *θ*(*T*).

$$\theta \left(T\right)=-5.4+1.8T-0.2124T^{2}+0.01015T^{3}-1.515\times 10^{-4}T^{4},$$ (3)

In order to ensure that the intrinsic rate *θ*(*T*) is positive, we assume the temperature interval of eq (3) is *T* ≥ 12°*C*. When *T* < 12°*C*, the intrinsic rate *θ*(*T*) is zero.

**The mortality rate of aquatic forms** *μ*_*a*_(*T*) **and adult mosquitoes** *μ*_*m*_(*T*).

$$\mu_{a}\left(T\right)=2.13-0.3797T+0.02457T^{2}-6.778\times 10^{-4}T^{3}+6.794\times 10^{-6}T^{4}.$$ (4)

$$\mu_{m}\left(T\right)=0.8692-0.1599T+0.01116T^{2}-3.408\times 10^{-4}T^{3}+3.809\times 10^{-6}T^{4}.$$ (5)

**The aquatic phase transition rate** *ε*(*T*).

$$\begin{array}{cc} \varepsilon (T)= & 0.131-0.05723T+0.01164T^{2}-0.001341T^{3}+0.00008723T^{4}\\ & -3.017\times 10^{-6}T^{5}+5.153\times 10^{-8}T^{6}-3.42\times 10^{-10}T^{7}. \end{array}$$ (6)

Eq (6) is used to describe the aquatic phase transition rate *ε*(*T*) over the temperature range of 10.54°*C* ≤ *T* ≤ 33.41°*C*. When *T* < 10.54°*C* or *T* > 33.41°*C*, *ε*(*T*) is zero.

**Daily biting rate** *b*(*T*). In the study of Scott et al. [39], a clear relationship between temperature and the blood feeding frequency *b*(*T*) was noted with the following equation:

b(T)=0.0043T+0.0943,21°C≤T≤32°C. (7)

Eq (7) shows the average daily biting rate *b*(*T*) increases gradually and linearly with *T* at the values from 0.18/day at *T* = 21°*C* to 0.23/day at *T* = 32°*C*. We extend this linear relationship down to a lower limit of 10.54°*C* for *Aedes albopictus* in Guangdong.

**Extrinsic incubation period** *σ*_*m*_(*T*). Enzyme kinetics model based on absolute reaction rate kinetics of enzyme was used to estimate the relationship between the extrinsic incubation period and temperature [40–42]. The dengue temperature-dependent extrinsic incubation rate is [33]:

$$\frac{1}{\sigma_{m}\left(T\right)}=24\times \frac{0.00333\times \frac{T_{k}}{298}\times exp\left(\frac{60513.2}{R}\left(\frac{1}{298}-\frac{1}{T_{k}}\right)\right)}{1+exp\left(\frac{705550}{R}\left(\frac{1}{308.352}-\frac{1}{T_{k}}\right)\right)},$$ (8)

where *T*_*k*_ = 273.15 + *T* is temperature in Kelvin and *R* is the Universal gas constant (1.987 *cal* *deg*^−1^ *mol*^−1^).

The descriptions of these six temperature-dependent parameters are listed in Table 2.

**Table 2:** Temperature-dependent parameters.

Notation	Description	Value	Reference
*θ*(*T*)	intrinsic oviposition rate of adult mosquito	Eq (3)	[25]
*μ*_*a*_(*T*)	mortality rate of aquatic mosquito	Eq (4)	[25]
*μ*_*m*_(*T*)	mortality rate of adult mosquito	Eq (5)	[25]
*ε*(*T*)	transition rate from aquatic into adult mosquito	Eq (6)	[25]
*b*(*T*)	mosquito biting rate	Eq (7)	[39]
*σ*_*m*_(*T*)	extrinsic incubation period of adult mosquito	Eq (8)	[33]

### Other model parameters

**Weekly temperature function**. The change of temperature in Guangzhou with respect to time is periodic and can be described by a Fourier function. Applying the MATLAB curve fitting toolbox for the real historical (from January 2013 to December 2014) temperatures in Guangzhou from related website, the first-order Fourier function can be written as,

T(t)=22.45-7.826cos(0.1179t)-2.007sin(0.1179t), (9)

And shown in Fig 5.

**Fig 5:**
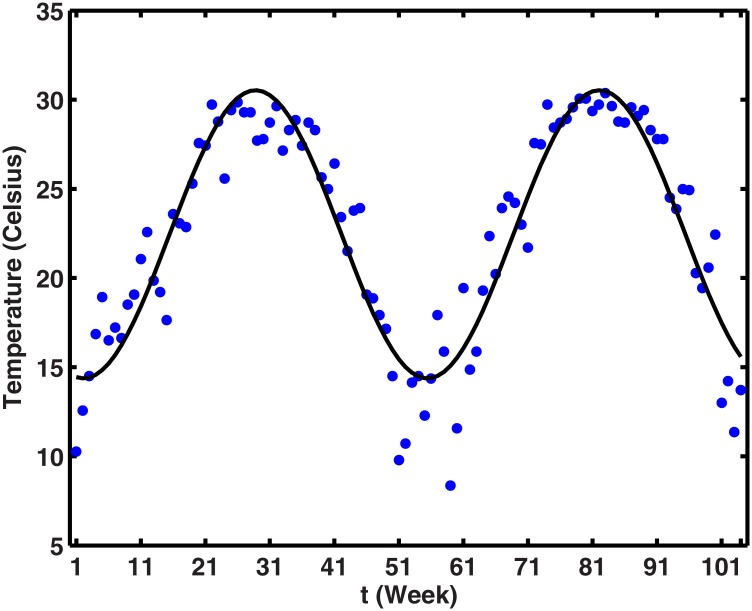
The comparison between the 2014 reported weekly average temperature and the simulated result with Eq (9). The blue dots indicate weekly average temperature in Guangzhou, while black line shows model output.

**The function of symptomatic imported dengue cases** (*B*(*t*)). According to previous description of model, the following equation can be used to describe the cumulative of symptomatic imported cases:

$$\frac{dZ(t)}{dt}=B\left(t\right).$$ (10)

**Fig 6:**
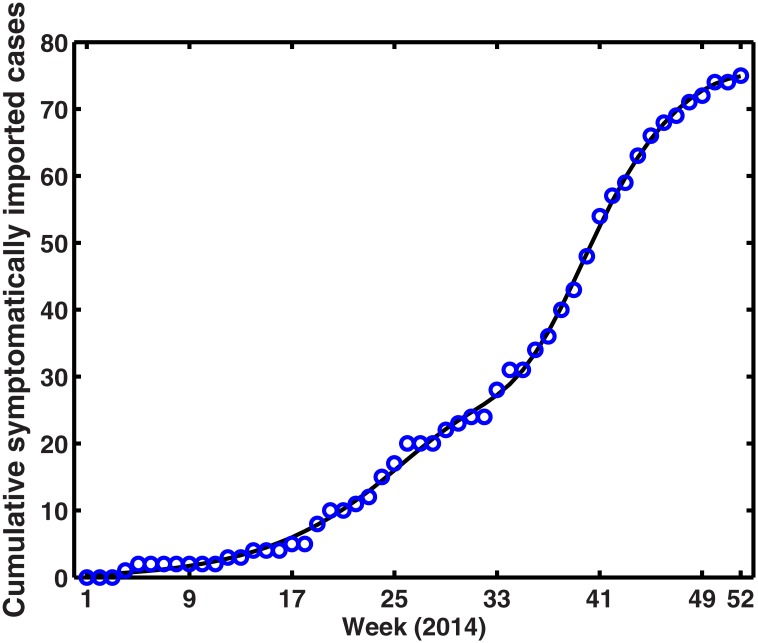
The weekly simulation result of cumulative symptomatically imported dengue cases with Eq (10) in 2014. The blue circles indicate the reporting weekly cumulative symptomatic imported dengue cases, while black line shows model output. The expression is $Z(t)=b_{1}*e^{-{\left(\frac{t-b_{2}}{b_{3}}\right)}^{2}}+b_{4}*e^{-{\left(\frac{t-b_{5}}{b_{6}}\right)}^{2}}$, where *b*_1_ = −9.252, *b*_2_ = 35.91, *b*_3_ = 5.854, *b*_4_ = 75.04, *b*_5_ = 52.94 and *b*_6_ = 22.61, respectively.

Weekly cumulative number of symptomatic imported dengue cases is carried out using a second-order Gaussian function by using the MATLAB curve fitting toolbox, shown in Fig 6. Hence, eq (11) can be used to describe the weekly imported dengue cases function.

$$B\left(t\right)=Z^{{}^{\prime }}\left(t\right)=-\frac{2}{b_{3}}*\frac{t-b_{2}}{b_{3}}*b_{1}*e^{-{\left(\frac{t-b_{2}}{b_{3}}\right)}^{2}}-\frac{2}{b_{6}}*\frac{t-b_{5}}{b_{6}}*b_{4}*e^{-{\left(\frac{t-b_{5}}{b_{6}}\right)}^{2}}.$$ (11)

**The inhibition rate of precipitation on larva** *κ*(*t*). Precipitation can change the water level in the environment. When the water level is higher, the environmental carrying capacity also increases; hence, the maximum number of mosquitoes the environment can support will also increase [43]. Hence, the amount of rainfall is associated with the mosquito population by increasing breeding sites or egg carrying capacity [27]. From Fig 3, we can obtain that the rainy season is from April until the end of August, and the high precipitation from May to August in the rainy season is abnormal in 2014. Hence, we used the following equation to describe the inhibition rate of precipitation on larva *κ*(*t*):

$$\kappa \left(t\right)=\kappa_{E}\left(\kappa_{m}+\left(1-\kappa_{m}\right)\sin^{2}\left(\frac{\pi t}{52}+\pi \right)\right),$$ (12)

where *κ*_*E*_ is the baseline carrying capacity and *κ*_*m*_ is the carrying capacity ratio between in dry and rainy seasons. We assume that the value of *κ*_*E*_ is associated with the weekly maximum rainfall whole year.

**Constant Parameters**. Several model parameters were available from the literature, and some were assumed. These are listed in Table 3.

**Table 3:** Some parameters description and values.

Notation	Description	Value	Range	Reference
*β*_*hm*_	transmission probability of human-to-vector	0.5	0.3-0.75	[44]
*β*_*mh*_	transmission probability of vector-to-human	0.4	0.1-0.75	[45]
*κ*_*m*_	the carrying capacity ratio between in dry and rainy seasons	0.2	0-1	Assumed
*σ*_*h*_	human latency period (day)	7	4-10	[2, 46–49]
*γ*_*h*_	duration of symptomatic infection (day)	7	4-10	[46, 47, 50]
*γ*_*y*_	duration of asymptomatic infection (day)	7	4-10	Assumed
*γ*_*w*_	duration of imported infection (day)	5	2-10	Assumed
*f*	fraction of female mosquitoes hatched from all eggs	0.5	0-1	[25, 33, 51, 52]
*k*	fraction of eggs hatching to larvae	0.5	0-1	Assumed
*p*	fraction of symptomatic infection	0.25	0-1	[3, 34]

## Results

### Estimation of parameters

On every Friday afternoon from September 24th to late November in 2014, the strategies for controlling mosquito vectors across many parts of Guangdong province were used [33]. Hence, the 2014 Guangdong dengue epidemic can be divided into three stages. the first stage is from the first week (January 1-5) to the 39th week (September 22-28) which can be assumed to have occurred without any intervention strategies; the second stage is from the 39th week (September 22-28) to the 48th week (November 24-30) and includes a major control effort (including clearing standing water and killing adult mosquitos); and, the third stage is from the start of December to the end of the year without any intervention strategies.

**Fig 7:**
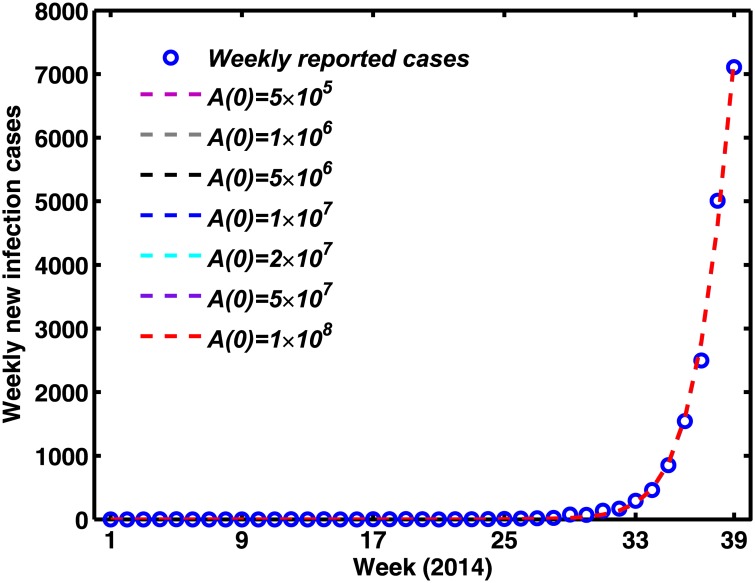
Sensitivity analysis on the initial value fitted to *κ*_*E*_ for each value of *A*(0).

Firstly, it is mainly to estimate the baseline of inhibition of precipitation on larva *κ*_*E*_ in eq (12). Because rates of infection have historically been so low (prior to the major outbreak) in Guangdong Province (see Table 1), the human population is assumed to be fully susceptible to the virus. Suppose that the initial value *S*_*h*_ equals to 1.0644 × 10^8^, which is the population for Guangdong province at the end of 2013 [53]. Assume that *A*(0) = 1 × 10^7^, and while all others (including *S*_*m*_(0), *E*_*m*_(0), *I*_*m*_(0), *E*_*h*_(0), *I*_*h*_(0), *Y*_*h*_(0), *W*_*h*_(0), *R*_*h*_(0)) are 0. The symptomatically reported dengue human cases on the bases of exponential growth for the first stage of 2014 were used to implement parameter estimation. We employed the adaptive Metropolis-Hastings algorithm to carry out extensive Markov-chain Monte-Carlo simulations [54, 55] for Eq (2) without any intervention strategies, and to estimate the mean and standard deviation value of the baseline of inhibition of precipitation on larva (*κ*_*E*_), which are 2.6042 × 10^6^ and 35746, respectively. We did not fit the initial value explicitly and performed a sensitivity analysis, changing values of *A*(0). Although various initial values of *A*(0) changed as *κ*_*E*_, it did not change the overall shape of the epidemic (Fig 7).

Then, we used the symptomatically reported dengue human cases from the 39th week to 48th week with the second stage of 2014 to implement parameter estimation (intervention parameters include the removal rate for adult mosquitos *α*_*m*_ and immature mosquitos *α*_*A*_). Here, suppose that *α*_*m*_ = *α*_*A*_. The adaptive Metropolis-Hastings algorithm was also used to carry out extensive Markov-chain Monte-Carlo (MCMC) simulations with Eq (2), and the mean and standard deviation value of *α*_*m*_, *α*_*A*_ are 0.8445 and 0.0134, respectively.

Finally, eq (2) without any intervention strategies was used to simulate the symptomatically reported dengue human cases from the 48th week to 52th week with the third stage of 2014.

### Fitting results

**Fig 8:**
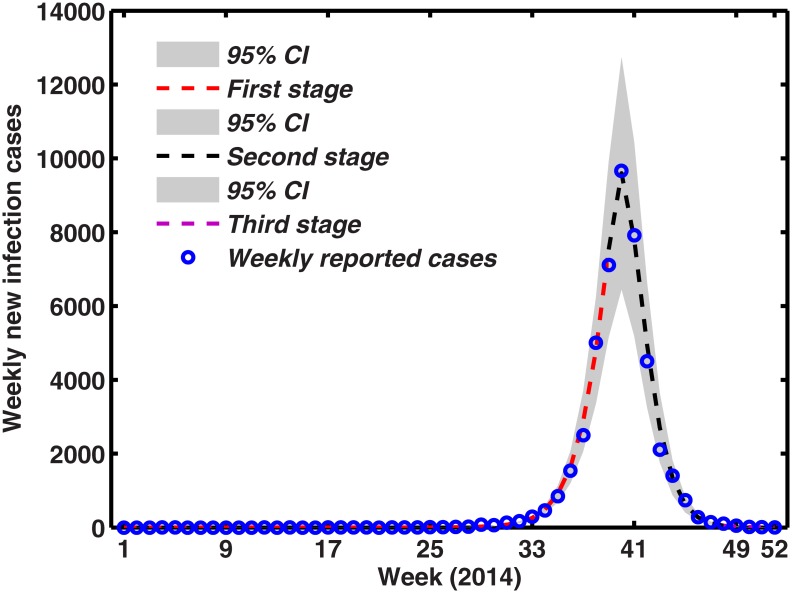
The simulation result of weekly new infection dengue cases in Guangdong in 2014. Blue circles indicate the number of weekly reported infection cases, light grey shaded area for the 95% confident interval (CI) for all 1000 simulations, while the red, blue and purple dotted lines are the median for all model outputs with the first, second and third stage, respectively. The first and third stage without any interventions, the second stage with interventions.

Through using the available model parameters from the literature in Tables 2 and 3, weekly temperature function in eq (9) and symptomatically imported dengue cases function in eq (11), Fig 8 unveils the time evolution of both infection cases and comparison with empirical record of dengue in Guangdong Province, and which also shows the 95% percent interval for all 1000 passing simulation trajectories and the median of these 1000 simulation outputs. It is clear that the theoretical prediction is nearly full agreement with real data, which also well validates the accuracy of proposed model.

### Driving force of the unprecedented outbreak of dengue in Guangdong, China

In Guangdong, dengue fever is still characterized as an imported epidemic disease and has not yet been confirmed to be endemic [9]. While in 2014, an unexpectedly large dengue epidemic was reported. Involving 45230 dengue fever cases, resulting in six deaths, exceeding the cumulative number of cases from 1990 to 2013. Reasons for this ongoing surge in dengue are currently unclear, so the possible causal factors for the 2014 unprecedented outbreak with different scenarios were investigated.

**Fig 9:**
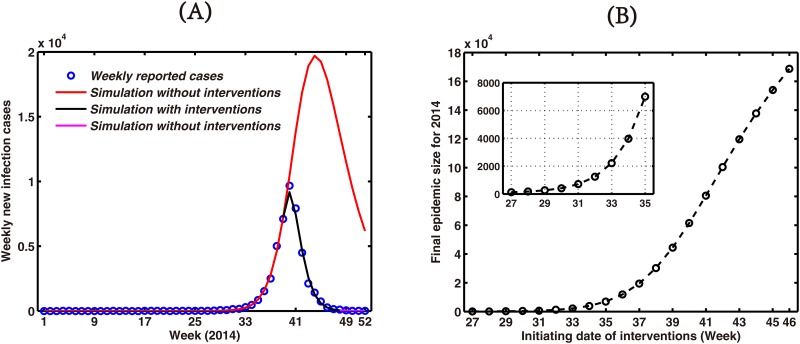
The comparison simulation result between with and without interventions. (A) Weekly new infection cases. (B) Simulations of the final outbreak size for 2014 with different initiating intervention dates.

Firstly, the impact of interventions on dengue epidemic was explored using our temperature-dependent parameters. Fig 9(A) shows the comparison simulations between with interventions from the 39th week (September 22-28) to 48th week (November 24-30) and without any interventions for weekly new infection cases. Intervention strategies can decrease the epidemic peak significantly for 2014 and prevent the disease spread to the more general population. Fig 9(B) shows the projected impact of initiating an equivalently conducted intervention with differing dates with the final outbreak size for 2014. Simulations demonstrate substantial gains that might be expected in implementing earlier control. For example, the final outbreak cases are reduced to fewer than 20,000 when interventions are simulated two weeks earlier i.e. from the 37th week (September 8-14) and to fewer than 10,000 when interventions are initiated from the 35th week (August 25-31).

**Fig 10:**
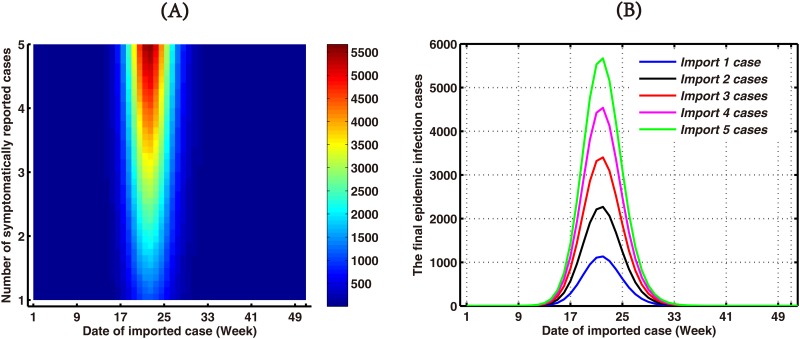
Simulation results for the final outbreak size of 2014 with different importation rates and dates. (A) Hot plot. (B) The final epidemic size for 2014 after changing the importation cases and dates. The blue line denotes the final epidemic size for 2014 after changing the date of 1 imported case. Black, red, purple and green lines are 2, 3, 4 and 5 imported cases, respectively.

Then, to explore the impact of imported cases, we investigated the final outbreak size of 2014 with different importation rates and dates, and recorded the final epidemic size (Fig 10). The final outbreak cases are anticipated to decrease as a direct proportion of the imported dengue cases (Fig 10(B)), and the dates and rates of imported case were crucial in producing the outbreak pattern in 2014. The figure show that when the imported case occurs in the 22nd week, the final epidemic size was the highest. And the 2014 unprecedented outbreak of dengue in Guangdong can not happen with only one imported cases, whenever the case is imported. From Fig 10, we can conclude that importations during the 17th week (April 21-27, 2014) to the 27th week (June 30–July 6, 2014) are the most likely to initiate autochthonous dengue outbreak, and the continuously imported cases is one causal factor for the 2014 unprecedented outbreak of dengue in Guangdong.

**Fig 11:**
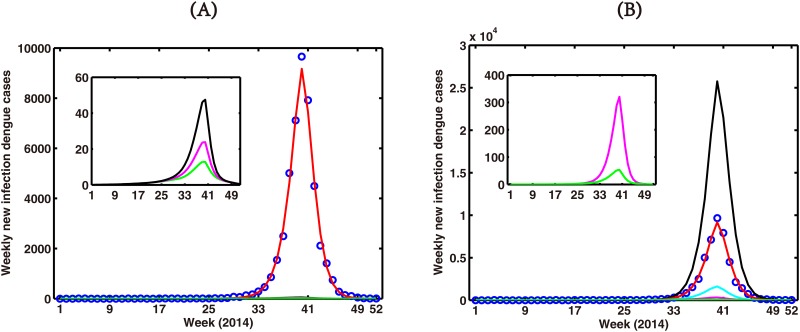
Trajectories of weekly new cases under different scenarios. Blue circles indicate the number of weekly reported infection cases. (A) Changing *η*. Red line denotes *η* = 100%. Black, purple and green lines are 20%, 10% and 0, respectively. (B) Changing *κ*_*E*_. The black line describes *κ*_*E*_ = 3.0 × 10^6^. And red, cyan, purple and green lines are 2.6042 × 10^6^, 2.0 × 10^6^, 1.5 × 10^6^, and 1.0 × 10^6^, respectively.

Finally, the impact of *η* and *κ*_*E*_ on weekly new cases were explored, where *η* is the proportion of the transmission of asymptomatic infections. Fig 11(A) shows that the 2014 unprecedented outbreak of dengue in Guangdong will not be occurred if asymptomatic dengue infections do not have transmission possibility or have low transmission possibility. Hence, asymptomatic dengue infections may be one possible causal factor for the unprecedented outbreak. To explore the relationship between precipitation and weekly new cases, the simulation results on different values of *κ*_*E*_ were shown in Fig 11(B). The outbreak peak cases are anticipated to decrease as a direct proportion of *κ*_*E*_ (the same as precipitation), so the high precipitation from May to August (which can provide more breeding sites and increased the environmental carrying capacity of mosquitos) may be another possible causal factor for the unprecedented outbreak.

## Discussion

In 2014, an unexpectedly large dengue epidemic was reported in Guangdong, involving 45230 cases and 76 imported cases in Guangdong province and exceeding the cumulative number of cases from 1990 to 2013, and this outbreak has posed a substantial socioeconomic burden. Facing up to the epidemic situation in Guangdong, the local government has been seeking forceful methods to reduce dengue transmission. Various prevention and control measures for vector control have been proposed by some researchers which include programs in community participation and health education to reduce mosquito breeding in household water containers [56]. No evidence yet exists to indicate this pathogen as endemic and local infections are attributed to imported cases [9]. From the reported cases (see Figs 1 and 2), the unprecedented outbreak of dengue in Guangdong province was result from the introduction of the virus by infected travelers from various areas of southeastern Asia where dengue is endemic. Here, a temperature-driven coupled entomological-epidemiological model was presented and assessed the role of seasonal vector dynamics and infection importation in driving dengue outbreaks. The model was also used to explore effective, local control and prevention measures.

Temperature, with its influence on the the extrinsic incubation period, adult mosquito mortality, immature stage developmental rates and bite rate, is an essential factor underlying dengue transmission. This has important implications in terms of the future epidemiology of dengue in China and a full assessment of this association is needed under different climate change scenarios.

Dengue virus infection in humans is often inapparent, and about 75% of all infectious are inapparent [3]. Our result shows that the 2014 unprecedented outbreak of dengue in Guangdong will not be occurred if asymptomatic dengue infections do not have transmission possibility or have low transmission possibility (See Fig 11(A)). It means that the large number of inapparent infections and subclinical cases occurred during the outbreak, which could greatly influence the transmission dynamics of dengue virus.

The intervention strategy has significant and long lasting effects on disease eradication, so the time of beginning intervention strategy will become more important. Simulating infection dynamics both pre- and post-interventions allowed an assessment of the approximate impact on reducing disease. Additionally, very substantial returns benefit from reducing delays in intervention following notification of local transmission (See Fig 9(B)). These results clearly demonstrate the improved early warning systems for this region of southern China is an urgent task.

Imported cases are the mainly predisposing factor for dengue transmission in Guangdong Province, China. Since we have the detailed information about the date and number of symptomatically imported cases, so eq (11) can be used to describe the weekly imported dengue cases function, and simulation result of cumulative symptomatically imported cases was shown in Fig 6. In terms of absolute numbers of imported cases, simulations suggested that the importance of this factor was overshadowed by their timing. Importations during the 17th week (April 21-27, 2014) to the 27th week (June 30–July 06, 2014) were the most likely to initiate autochthonous dengue outbreak, and a case imported around the 22nd week (May 26–Jun 01, 2014) appears to have triggered the biggest outbreak in 2014, which is different with paper [33] (See Fig 10). Identification of this critical window should enable efforts in surveillance and prevention to focus on when identifying imported infections is most important to local public health.

In 2015, there existed a little outbreak with 1547 autochthonous cases and 153 imported cases in Guangdong. Although there are more imported cases than 2014, the possible factors for little dengue outbreak in 2015 may be the early mosquito control (started in April), early detection and quarantine of imported cases [33]. Moreover, there are many reports on dengue in media and network after the unprecedented outbreak in 2014, so residents could pay more attention to the information on dengue and know how to avoid the transmission, this may be another factor.

The current study suffers from several limitations. As with all models, the cost of transparency and simplicity of our model is realism and our model does not include spatial effects but instead treats Guangdong province as a homogenous and well-mixed population. Targeting specific sub-populations with control may be achievable more rapidly and therefore a spatial age-structured model would be anticipated as an important tool to expedite intervention. Additionally, data are based on passive case surveillance and hence only apply to the symptomatic proportion of infected individuals when the proportion of asymptomatic individuals can be substantial and variable [57]. If data became available from active surveillance to identify this proportion (and ascertain whether and how it varies over the time-course of an epidemic), this information could easily be incorporated in the model as an additional epidemiological compartment. Nevertheless, model fitting to data was generally good and derived parameterizations were biologically intuitive, lending confidence to our outputs and justifying the model’s further use and development for future analysis.

## References

[1] Health topics: Dengue. World Health Organization. Available: http://www.who.int/topics/dengue/en/.

[2] WHO. Dengue: guidelines for diagnosis, treatment, prevention and control. World Health Organization; 2009. 23762963

[3] Bhatt S, Gething PW, Brady OJ, Messina JP, Farlow AW, Moyes CL, et al The global distribution and burden of dengue. Nature. 2013; 496(7446): 504–507. 10.1038/nature12060 23563266 PMC3651993

[4] WHO. Global strategy for dengue prevention and control 2012–2020. World Health Organization; 2012.

[5] Halloran ME, Longini IM Jr. Emerging, evolving, and established infectious diseases and interventions. Science. 2014; 345: 1292–1294. 10.1126/science.1254166 25214617 PMC4408765

[6] Jones KE, Patel NG, Levy MA, Storeygard A, Balk D, Gittleman JL, et al Global trends in emerging infectious diseases. Nature. 2008; 451(7181): 990–993. 10.1038/nature06536 18288193 PMC5960580

[7] Zhao HL, Luo QH, Shen G. The epidemic of dengue fever at Shiwanzhen of Foshan city in 1978 (in Chinese). Natl Med J China. 1981; 61: 366–369. 6800615

[8] Lai S, Huang Z, Zhou H, Anders KL, Perkins TA, Yin W, et al The changing epidemiology of dengue in China, 1990–2014: a descriptive analysis of 25 years of nationwide surveillance data. BMC Med. 2015; 13(1): 1 10.1186/s12916-015-0336-1 25925417 PMC4431043

[9] Wu JY, Lun ZR, James AA, Chen XG. Dengue fever in mainland China. Am J Trop Med Hyg. 2010; 83: 664–671. 10.4269/ajtmh.2010.09-0755 20810836 PMC2929067

[10] Jin LQ, Li D. A recent survey of mosquito fauna in Guangdong Province, southern China, with a review of past records. Med Vet Entomol. 2008; 22(4): 359–363. 10.1111/j.1365-2915.2008.00758.x 19120964

[11] Zhao H, Zhao L, Jiang T, Li X, Fan H, Hong W, et al Isolation and characterization of dengue virus serotype 2 from the large dengue outbreak in Guangdong, China in 2014. Science China Life Sciences. 2014; 57(12): 1149–1155. 10.1007/s11427-014-4782-3 25502398 PMC7089550

[12] Jiang L, Wu X, Wu Y, Bai Z, Jing Q, Luo L, et al Molecular epidemiological and virological study of dengue virus infections in Guangzhou, China, during 2001–2010. Virol J. 2013; 10(1): 1 10.1186/1743-422X-10-4 23282129 PMC3558415

[13] He JF. Dengue fever surveillance and prevention and control in guangdong province. The centers for disease control and prevention of guangdong province; 2014.

[14] Sang S, Chen B, Wu H, Yang Z, Di B, Wang L, et al Dengue is still an imported disease in China: A case study in Guangzhou. Infection, Genetics and Evolution. 2015; 32: 178–190. 10.1016/j.meegid.2015.03.005 25772205

[15] Sun GQ. Mathematical modeling of population dynamics with Allee effect. Nonlinear Dynam. 2016; 85: 1–12. 10.1007/s11071-016-2671-y

[16] Sun GQ, Wu ZY, Jin Z, Wang Z. Influence of isolation degree of spatial patterns on persistence of populations. Nonlinear Dynam. 2016; 83: 811–819. 10.1007/s11071-015-2369-6

[17] Sun GQ, Wang SL, Ren Q, Jin Z, Wu YP. Effects of time delay and space on herbivore dynamics: linking inducible defenses of plants to herbivore outbreak, Sci Rep. 2015; 5: 11246 10.1038/srep11246 26084812 PMC4471659

[18] Jetten TH, Focks DA. Potential change in the distribution of dengue transmission under climate warming. Am J Trop Med Hyg. 1997; 57: 285–297. 9311638 10.4269/ajtmh.1997.57.285

[19] Khasnis AA, Nettleman M. Global warming and infectious disease. Arch Med Res. 2005; 36: 689–696. 10.1016/j.arcmed.2005.03.041 16216650

[20] Esteva L, Yang HM. Mathematical model to assess the control of *Aedes aegypti* mosquitoes by the sterile insect technique. Math Biosci. 2005; 198: 132–147. 10.1016/j.mbs.2005.06.004 16125739

[21] Ferreira CP, Yang HM, Esteva L. Assessing the suitability of sterile insect technique applied to *Aedes aegypti*. J Biol Syst. 2008; 16: 1–13. 10.1142/S0218339008002691

[22] Yang HM, Ferreira CP. Assessing the effects of vector control on dengue transmission. Appl Math Comput. 2008; 198: 401–413. 10.1016/j.amc.2007.08.046

[23] Cailly P, Tran A, Balenghien T, L’Ambert G, Toty C, Ezanno P. A climate-driven abundance model to assess mosquito control strategies. Ecol Model. 2012; 227: 7–17. 10.1016/j.ecolmodel.2011.10.027

[24] Morin CW, Monaghan AJ, Hayden MH, Barrera R, Ernst K. Meteorologically driven simulations of dengue epidemics in San Juan, PR. PLoS Negl Trop Dis. 2015; 9(8): e0004002 10.1371/journal.pntd.0004002 26275146 PMC4537107

[25] Yang HM, Macoris MLG, Galvani KC, Andrighetti MTM, Wanderley DMV. Assessing the effects of temperature on the population of *Aedes aegypti*, the vector of dengue. Epidemiol Infect. 2009; 137: 1188–202. 10.1017/S0950268809002052 19192322

[26] Liu-Helmersson J, Stenlund H, Wilder-Smith A, Rocklov J. Vectorial capacity of *Aedes aegypti*: effects of temperature and implications for global dengue epidemic potential. PLoS ONE. 2014; 9: e89783 10.1371/journal.pone.0089783 24603439 PMC3946027

[27] Polwiang S. The seasonal reproduction number of dengue fever impacts of climate on transmission. PeerJ PrePrints. 2015; e1142 10.7717/peerj.1069 26213648 PMC4512769

[28] Pinho STRD, Ferreira CP, Esteva L, Barreto FR, Silva VM, Teixeira MGL. Modelling the dynamics of dengue real epidemics. Phil Trans R Soc A. 2010; 368: 5679–5693. 10.1098/rsta.2010.0278 21078642

[29] Lourenco J, Recker M. The 2012 Madeira Dengue Outbreak: Epidemiological Determinants and Future Epidemic Potential. PLoS Negl Trop Dis. 2014; 8: e3083 10.1371/journal.pntd.0003083 25144749 PMC4140668

[30] Wesolowski A, Qureshi T, Boni MF, Sundsøy PR, Johansson MA, Rasheed SB, et al Impact of human mobility on the emergence of dengue epidemics in Pakistan. Proc Natl Acad Sci USA. 2015; 112(38): 11887–11892. 10.1073/pnas.1504964112 26351662 PMC4586847

[31] Sang S, Yin W, Bi P, Zhang H, Wang C, Liu X, et al Predicting local dengue transmission in Guangzhou, China, through the influence of imported cases, mosquito density and climate variability. PLoS One. 2014; 9(7): e102755 10.1371/journal.pone.0102755 25019967 PMC4097061

[32] Sang S, Gu S, Bi P, Yang W, Yang Z, Xu L, et al Predicting unprecedented dengue outbreak using imported cases and climatic factors in Guangzhou, 2014. PLoS Negl Trop Dis. 2015; 9(5): e0003808 10.1371/journal.pntd.0003808 26020627 PMC4447292

[33] Cheng Q, Jing Q, Spear RC, Marshall JM, Yang Z, Gong P. Climate and the timing of imported cases as determinants of the dengue outbreak in Guangzhou, 2014: evidence from a mathematical model. PLoS Negl Trop Dis. 2016; 10(2): e0004417 10.1371/journal.pntd.0004417 26863623 PMC4749339

[34] Zhu G, Liu J, Tan Q, Shi B. Inferring the spatio-temporal patterns of dengue transmission from surveillance data in Guangzhou, China. PLoS Negl Trop Dis. 2016; 10(4): e0004633 10.1371/journal.pntd.0004633 27105350 PMC4841561

[35] Chastel C. Eventual role of asymptomatic cases of dengue for the introduction and spread of dengue viruses in non-endemic regions. Front Physiol. 2012; 3: 70 10.3389/fphys.2012.00070 22479252 PMC3315825

[36] Stolerman LM, Coombs D, Boatto S. SIR-Network model and its application to dengue fever. SIAM J Appl Math. 2015; 75(6): 2581–2609. 10.1137/140996148

[37] Wang T, Wang M, Shu B, Chen XQ, Luo L, Wang J, et al Evaluation of inapparent dengue infections during an outbreak in southern China. PLoS Negl Trop Dis. 2015; 9(3): e0003677 10.1371/journal.pntd.0003677 25826297 PMC4380470

[38] World Health Organization. Situation update of dengue in the SEA Region, 2010. Geneva: World Health Organization; 2011.

[39] Scott TW, et al Longitudinal studies of *Aedes aegypti* (Diptera: Culicidae) in Thailand and Puerto Rico: blood feeding frequency. J Med Entomol. 2000; 37: 89–101. 10.1603/0022-2585-37.1.89 15218911

[40] Sharpe PJ, DeMichele DW. Reaction kinetics of poikilotherm development. J Theor Biol. 1977; 64(4): 649–670. 10.1016/0022-5193(77)90265-X 846210

[41] Schoolfield RM, Sharpe PJ, Magnuson CE. Non-linear regression of biological temperature-dependent rate models based on absolute reaction-rate theory. J Theor Biol. 1981; 88: 719–731. 10.1016/0022-5193(81)90246-0 6790878

[42] Otero M, Solari HG, Schweigmann N. A stochastic population dynamics model for *Aedes aegypti*: formulation and application to a city with temperate climate. Bul Math Biol. 2006; 68: 1945–1974. 10.1007/s11538-006-9067-y 16832731

[43] Karl S, Halder N, Kelso JK, Ritchie SA, Milne GJ. A spatial simulation model for dengue virus infection in urban areas. BMC Infect Dis. 2014; 14: 447 10.1186/1471-2334-14-447 25139524 PMC4152583

[44] Chikaki E, Ishikawa H. A dengue transmission model in Thailand considering sequential infections with all four serotypes, J Infect Dev Countr. 2009; 3(9): 711–722. 10.3855/jidc.616 19858573

[45] Andraud M, Hens N, Marais C, Beutels P. Dynamic epidemiological models for dengue transmission: a systematic review of structural approaches, PLoS One 2012; 7(11): e49085 10.1371/journal.pone.0049085 23139836 PMC3490912

[46] Massad E, Coutinho FA, Lopez LF, da Silva DR. Modeling the impact of global warming on vector-borne infections. Phys Life Rev. 2011; 8: 169–199. 10.1016/j.plrev.2011.01.001 21257353

[47] Amaku M, Coutinho FAB, Raimundo SM, Lopez LF, Burattini MN, Massad E. A comparative analysis of the relative efficacy of vector-control strategies against dengue fever. Bul Math Biol. 2014; 76: 697–717. 10.1007/s11538-014-9939-5 24619807

[48] Burattini MN, Chen M, Chow A, Coutinho FAB, Goh KT, Lopez LF, et al Modelling the control strategies against dengue in Singapore. Epidemiol Infect. 2008; 136(03): 309–319. 10.1017/S0950268807008667 17540051 PMC2870819

[49] Chan M, Johansson MA. The incubation periods of dengue viruses. PLoS ONE. 2012; 7: e50972 10.1371/journal.pone.0050972 23226436 PMC3511440

[50] Halstead SB. Dengue In Warren KS, Mahmoud AAF, editors. Tropical and geographical medicine. New York: McGraw-Hill; 1990 pp. 675–684.

[51] Delatte H, Gimonneau G, Triboire A, Fontenille D. Influence of temperature on immature development, survival, longevity, fecundity, and gonotrophic cycles of *Aedes albopictus*, vector of chikungunya and dengue in the Indian Ocean. J Med Entomol. 2009; 46(1): 33–41. 10.1603/033.046.0105 19198515

[52] Tran A, L’Ambert G, Lacour G, Benoît R, Demarchi M, Cros M, et al A rainfall-and temperature-driven abundance model for *Aedes albopictus* populations. Int J Env Res Pub He. 2013; 10(5): 1698–1719. 10.3390/ijerph10051698 23624579 PMC3709343

[53] China population & employment statistics yearbook. Beijing: China Statistics Press; 2014.

[54] Haario H, Laine M, Mira A, Saksman E. Dram: Efficient adaptive mcmc. Stat Comput. 2006; 16: 339–354. 10.1007/s11222-006-9438-0

[55] Gamerman D, Lopes HF. Markov chain monto carlo: stochastic simulation for bayesian inference. 2nd ed Taylor and Francis Group, London New York; 2006.

[56] Wu Y, Zheng X, Wu Z. Dengue Fever in China Treatment of human parasitosis in traditional Chinese medicine. Springer Berlin Heidelberg; 2014 pp. 239–253. 10.1007/978-3-642-39824-7_15

[57] Endy TP, Anderson KB, Nisalak A, Yoon IK, Green S, Rothman AL, et al Determinants of inapparent and symptomatic dengue infection in a prospective study of primary school children in Kamphaeng Phet, Thailand. PLoS Negl Trop Dis. 2011; 5(3): e975 10.1371/journal.pntd.0000975 21390158 PMC3046956

